# Why visiting one’s ageing mother is not enough: on filial duties to prevent and alleviate parental loneliness

**DOI:** 10.1007/s11019-020-10000-5

**Published:** 2021-01-08

**Authors:** Bouke de Vries

**Affiliations:** 1 Department of Historical, Philosophical and Religious Studies, Umeå University, Umeå, Sweden; 2 Centre for Biomedical Ethics and Law, KU Leuven, Leuven, Belgium

**Keywords:** Loneliness, Social isolation, Filial duties, Children, Parents, Family ethics, Parent- child relations

## Abstract

As people grow old, many risk becoming chronically lonely which is associated with e.g. depression, dementia, and increased mortality. Whoever else should help to protect them from this risk, various philosophers have argued that any children that they might have will often be among them. Proceeding on this assumption, this article considers what filial duties to protect ageing parents from loneliness consist of, or might consist of. I develop my answer by showing that a view that may be intuitively plausible, namely that they simply require children to visit their ageing parents regularly when they can do so at reasonable cost and call, text, and/or email them from time to time, is defective in three respects. First, it ignores children’s potential responsibilities to encourage and/or facilitate social interaction between their parents and third parties. Second, it ignores their potential responsibilities to help provide their parents with non-human companionship. Third, it elides over their duties to coordinate their efforts to offer loneliness protection with others. What I end up proposing instead, then, is an approach for protecting ageing parents from loneliness that is multi-faceted.

## Introduction

As people grow old, a large proportion risks becoming chronically lonely. Not only do most experience a substantial reduction in the size of their social network, with some studies suggesting that the average social network of those aged 85 and above is nearly half of that of 70–84 year olds (Lang and Carstensen 1994), for many members of this group, the death of romantic partners, friends, and siblings means that they lose their main confidants. Compensating for these losses is often difficult, moreover, as the ability of many older adults to maintain existing social contacts and create new ones is compromised by the onset of age-related disabilities and illnesses (Dykstra et al. 2005; Willis et al. 2019), as well as by the existence of negative stereotypes about the capacity of older individuals to make valuable contributions to relationships, especially those with dementia (Shiovitz-Ezra et al. 2018; Hope 2010). It should not come as a surprise, therefore, that studies from Europe and North-American report that circa 20 to 35 percent of adults between the ages of 65 and 79 say that they are often lonely, a figure that raises to 40 to 50 percent among those aged 80 and above (Dykstra 2009).

The fact that ageing individuals are highly vulnerable to chronic loneliness is a serious problem as this type of loneliness has been found to contribute to a range of negative health outcomes, including depression (Cacioppo et al. 2010; Victor and Yang 2012); dementia (Holwerda et al. 2012); alcoholism (Åkerlind and Hörnquist 1992); increased mortality (Luo et al. 2012); and poor physical health (Aanes et al. 2010), with some experts arguing that its health effects can be compared to that of smoking 15 cigarettes a day (Novotney 2019). As such, there are good grounds for thinking that even if the majority of adults can be expected to protect themselves from loneliness during their early and middle life-stages, a period during which many still have what Kimberley Brownlee (2013) terms “adequate opportunities for decent or supportive social contact”, this will change in many cases as they grow old. Even if most older adults retain at least some responsibility for ensuring that they do not become lonely or stay lonely depending on the level of autonomy of which they remain capable, the social losses that they experience along with the abovementioned difficulties that many of them face in maintaining existing relationships and forging new ones suggest that this responsibility will usually be shared with others.

Proceeding on the assumption that this is correct, this article considers how one particular group should help protect older adults from loneliness, namely adult children. The reason for focusing upon this group is that there exists a consensus within the philosophical literature that many adult children have special moral duties to look after their parents’ well-being, i.e. duties that most other people lack. These duties might derive from the fact that they have a loving relationship with their parents (Mills 2003; English 1992; Dixon 1995); the fact that they owe their parents gratitude for their upbringing (Berger 1975); the fact that existing social norms have raised legitimate expectations of filial support among their parents (Sommers 1986); and/or the fact that interacting with their parents provides the parents with special goods, such as a sense of intergenerational continuity (Keller 2006). What is important for this article’s purposes is that insofar as filial duties to look after the wellbeing of ageing parents are indeed common, as I assume here they are, then it seems that, *whoever else* should help protect older people from chronic loneliness, any adult children that they might have will often be among them, and some philosophers have defended this claim (e.g. Schinkel 2012, p. 414; de Vries 2020).

Now, the answer to the question of what filial duties to protect ageing parents from loneliness require might seem obvious. Since loneliness consists of a disutility-inducing discrepancy between one’s realized relationships and the types of relationships that one desires (more on this below), and since most parents value their relationships with their children, it may appear that they simply require children to visit their parents regularly when they can do so at reasonable cost^1^ and call, text, and/or email them from time to time. Call this the ‘just visit and stay in touch’ view for short.

As intuitive as the ‘just visit and stay in touch’ view might seem, I argue in this article that it is mistaken. Whilst it is undeniably important for children to visit their ageing parents and to stay in touch with them through texts, phone calls, and/or email exchanges, there are three problems with the notion that this is *all that* filial duties to prevent and alleviate parental loneliness require, or can require. First, it ignores children’s potential responsibilities to encourage and/or facilitate social interaction between their parents and third parties. Second, it ignores their potential responsibilities to help provide their parents with non-human companionship. Third, it elides over their duties to coordinate their efforts to offer loneliness protection with others. What I end up proposing instead, then, is an approach for protecting ageing parents from loneliness that is multi-faceted.

Before vindicating these claims, a few clarifications are in order. When speaking of ‘parents’, I am referring to individuals who at some stage of their lives not only have a legal responsibility for nurturing a specific minor to whom they may or may not be biologically related, but who also play an important role in that minor’s upbringing. For people to be ‘ageing’, ‘old’, or—as is more politically correct to say—‘older’ here means that they have reached a minimum age of approximately 65 years,^2^ whereas ‘loneliness’ can be defined in more detail as a state.

[W]here there is an unpleasant or inadmissible lack of certain relationships. This includes situations in which the number of existing relationships is smaller than is considered desirable, as well as situations where the intimacy one wishes for has not been realized (de Jong-Gierveld 1987, p. 120).

## Three problems with the ‘just visit and stay in touch’ view

With these definitions in the background, let us examine in more detail the problems with what I have termed the ‘just visit and stay in touch’ view according to which it is necessary and sufficient for the fulfilment of filial duties to prevent and alleviate parental loneliness that children visit their ageing parents regularly when they can do so at reasonable cost and call, text, and/or email them from time to time.

### Child-parent relationship bias

A first problem with this view is that it ignores children’s potential duties to encourage and/or facilitate social interaction between their ageing parents and *third parties*. One context where such duties often exist is when ageing parents are ill-prepared for the inevitable reductions in the size of their social network towards the end of their life. A common example of this is when someone has a romantic partner but few friends, or simply few close friends. To guard against such losses, i.e. to ensure that people are left with a social network in the final stages of their life that, although significantly smaller than it once was, still does a reasonably good job in protecting them from loneliness, De Jong-Gierveld and Fokkema (2015) have argued that it is imperative that they build resilient social networks *before* they reach an age where it becomes too difficult for them to do so as a result of mobility impairments and age-related diseases. Such networks contain ties to a sufficiently large number of individuals, but ideally also intimate relationships with more than one person given that the loss of a romantic partner has been found to be a major determinant of loneliness among older adults (Jones et al. 1985; Golden et al. 2009; Dahlberg et al. 2015).

What is pertinent for us is that, if a resilient social network is indeed one of the best safeguards against loneliness in old age, if not the best safeguard as De Jong-Gierveld and Fokkema argue, then it is plausible that many children with duties to protect their ageing parents from loneliness will need to encourage their parents to establish or maintain such networks and/or assist them in doing so. This may require, for instance, that they

- Alert their parents to the fact that having a small social network leaves them vulnerable to loneliness.
- Alert their parents to the fact that chronic loneliness contributes to a range of serious mental and physical health problems (see the introduction).
- Help their parents to meet new people. This could be done, for instance, by helping them to get to social events on foot, by car, or by public transport. Another possible measure for those who can afford it is to gift membership of a social organization (e.g. a choir or sports club) to their parents.
  And/or
- Help their parents to preserve or strengthen any existing relationships with third parties that they might have. Ways of doing so may include, but are not limited to, driving a parent to a friend whom she is no longer able to visit independently; baby-sitting one’s cousin so that one’s sibling can visit one’s parent(s) during the evening; and paying for public transport tickets that allow one’s own children to visit one’s parent(s) after school.

I should add here that *even when* ageing parents are so well-connected that losing some of their relationships does not deprive of them a resilient social network, for their children to help them sustain certain relationships with third parties might still be important, if not necessary, in order to protect them from loneliness. The reason for this lies in the fact that we do not simply care about our relationships because we want to have a sufficient number of them. We also care about them because we value them for their own sake, or at least any good relationships that we may have, which means that the loss of, say, a specific friendship might cause us to feel lonely even if we retain several close friends.

Another thing to mention is that, in saying that some children ought to encourage and/or facilitate social interaction between their ageing parents and third parties, I do not mean to suggest that this is *all that* their duties to help prevent and alleviate parental loneliness will normally require of them. Given the emotional attachments that most parents have to their children, for children to socially interact with their ageing parents, whether privately or in the company of others, will usually be necessary as well in order to effectively protect the latter from loneliness.

### Human companionship bias

A second problem with the ‘just visit and stay in touch’ view is that it assumes that the provision of access to human companionship is the only way, or only appropriate way, of protecting people from loneliness. In so doing, it ignores that filial duties to help prevent and alleviate parental loneliness may, and sometimes will, require children to make *non-human companionship* available to their parents or to simply assist in making such companionship available to them. Non-human companionship might be offered by pets, which have been found to reduce feelings of loneliness in several studies (e.g. Krause-Parello et al. 2014; Banks and Banks 2002; Powell et al. 2019). However, it might also be offered by social robots whose capacity for reducing loneliness has been similarly corroborated by empirical research (e.g. Banks et al. 2008; Kanamori et al. 2002; Mordoch et al. 2013; Barrett et al. 2017). Prominent examples of such robots include the therapeutic seal Paro (Dapin 2019), which can e.g. recognize its name, greetings, and praise, and the humanoid robot Pepper, which has the ability to e.g. recognize faces and read basic human emotions (Aaltonen et al. 2017).

There are different ways in which children may help to make non-human companionship available to their parents. One is for them to contribute financially to the purchase of a pet or a social robot if they can afford to do so. Other ones are for them to assist in the care that pets require, for example by walking a parent’s dog, or to assist in the maintenance that social robots require, for example by updating the software of a parent’s robot every few months. To see why some children ought to do such things in order to discharge their duties to protect their ageing parents from loneliness, it should be noted that, unlike most humans that people might socially interact with, pets and robots are available for social interaction *most of the time*. This high level of availability is of significant value when individuals have few regular visitors and little access to human sociability more generally, which is common among old people in countries that are struggling to provide decent care to their older populations. For example, in a recent study, health economist Heinz Rothgang (2020) found that residents of German nursing homes currently receive a daily average of 99 min of care whereas they are estimated to require a daily average of 141 min in order to enjoy a minimally decent living standard.

Lest I be misunderstood, I am not claiming that having a pet or a social robot is a solution for *everyone’s* loneliness or for *everyone’*s risk of becoming lonely. Some of us are allergic to pets, whereas others simply do not want the companionship of either a pet or a social robot (Vandemeulebroucke et al. 2019). With respect to social robots specifically, another problem that may exist is that users with advanced dementia do not realize that they are interacting with a non-sentient entity (Vandemeulebroucke et al. 2018, p. 19). Rather than claiming that *all* children with duties to protect their ageing parents from loneliness should help to provide their parents with non-human companionship, then, my contention is more modest, namely that *some of them* ought to do so in order to discharge these duties.

At this point, a critic might argue that helping to make non-human companionship available is the *wrong way* for children to protect their ageing parents from loneliness even if the parents are happy to accept this solution. On this view, respecting people’s dignity requires that their children protect them from loneliness by providing them with access to *human companionship*, whereby at least some of this companionship should consist of the children’s *own companionship* as opposed to the companionship of third parties (see the previous subsection).

There are two things to be said in response. The first concerns a point I made earlier, namely that because of the emotional attachments that parents generally have to their children, for children to visit their ageing parents and call, text, and/or email them from time to time is likely to remain necessary in most cases in order to effectively prevent and alleviate parental loneliness. As such, cases where children can wholly discharge their duties to protect their ageing parents from loneliness by helping to provide them with non-human companionship are bound to be rare, if they exist at all.

Our critic might retort that, even if this is so, the mere fact that helping to provide non-human companionship *could potentially suffice* to discharge filial duties to prevent and alleviate parental loneliness remains counterintuitive. This view is predicated upon the notion that, as long as parents have not disowned or seriously neglected or abused their children, it would be an affront to their dignity if their children never visited them or tried to stay in touch through phone calls, texts, and/or email exchanges.

The problem with view, and this brings me to the second response, lies in its assumption that if duties to prevent and alleviate parental loneliness do not require children to socially interact with their parents, then they *cannot* have moral duties to engage in such interaction. This, of course, is not true. Even in the unlikely event that children are able to wholly fulfil their duties to protect their ageing parents from loneliness by helping to make non-human companionship available to them, they might still have moral duties to socially interact with their parents, which may be because this is necessary in order to respect their parents’ dignity (as the current objection assumes) and/or because of some other reason, such as that socializing with their parents serves their parents’ autonomy.

### Too-many-cooks problem

A third and final problem with the ‘just visit and stay in touch’ view might be labelled the ‘too many cooks’ problem. According to this problem, just as having several cooks prepare a soup without coordination is likely to result in a suboptimal mix of flavors as each cook is likely to add her preferred ingredients without due regard to what the other cooks have added or will subsequently add, so older adults are likely to end up being sub-optimally protected from loneliness when those who seek to offer them loneliness protection^3^ fail to coordinate their efforts. To bring this out, it should be noted that there are various ways in which such coordination can, and often does, enable people to offer better loneliness protection than if each of them were to act on their own accord, which is something which the ‘just visit and stay in touch’ view allows them to do.

- *Non-overlapping visits* For starters, coordination may help to avoid situations whereby someone is visited by multiple individuals simultaneously when he would be better protected from loneliness if each visitor were to visit him at a different time. Suppose that a person A has two friends, B and C, who are not mutual friends and who in fact profoundly dislike each other. Under these conditions, it might bring much more joy to A and, consequently, better loneliness protection, if B’s and C’s visits do not overlap given the damping effect on the atmosphere that their joint presence is likely to have.
- *Overlapping visits* Coordination may also be used to achieve the opposite result by ensuring that the visits of certain individuals overlap. There are cases where such overlapping visits better protect the visitee(s) from loneliness than separate visits. For some parents, for instance, receiving joint visits from their children might be more rewarding, and therefore provide them with better protection against loneliness, than receiving separate visits from their children. This is because whilst separate visits entail more frequent visits, such visits do not have the potential added value of a family reunion.
- *Visits at regular intervals* Still another way in which coordination may secure better loneliness protection is that it helps to facilitate a more even spread of visits. Such a spread will be important for those who are less likely to become lonely when they are visited at regular intervals as opposed to having periods with relatively many visits followed by periods with relatively few visits during which they are particularly prone to loneliness.

Besides coordinating the provision of human companionship, children with duties to protect their ageing parents from loneliness may, and sometimes will, need to coordinate *the provision of non-human companionship* to their parents. Consider a case where having the company of a relatively expensive social robot is likely to make an older parent less lonely, but where neither she nor her children can personally afford this robot. Under these conditions, it might be morally necessary for her children to collectively raise money for the robot, which will require them to coordinate their actions.

In other cases, coordination will be required for ensuring parents’ *continued enjoyment of non-human companionship*. Suppose that an older parent can no longer walk his dog and that he would become very lonely if he had to give his dog away. Under these conditions, it might be incumbent upon his children to walk his dog for him. However, when none of the children have time to walk his dog on a daily basis, a schedule may need to be agreed that specifies which child will walk the dog on which day of the week.

## A different approach for preventing and alleviating parental loneliness

In criticizing the ‘just visit and stay in touch’ view, a multi-faceted approach for discharging filial duties to protect ageing parents from loneliness has emerged. Whilst it remains necessary on this approach for most, if not all, children with such duties to socially interact with their parents, it requires many of them to spend part of their time and resources on providing other forms of loneliness protection in order to offer better protection overall. As I have argued, this may involve encouraging and/or facilitating social interaction between their parents and third parties; helping to make non-human companionship available to their parents; and coordinating their efforts to protect their parents from loneliness with others.

I want to end with two comments. The first is that whereas I have assumed that a proportion of children has moral duties to protect their ageing parents from loneliness, the fact that the proposed approach offers more effective loneliness protection than the ‘just visit and stay in touch’ approach means that there are moral reasons for favoring it over the latter *even if* no child ever had such duties.

The second comment is that, although I have focused on filial duties to protect ageing parents from loneliness, children are by no means the *only agents* who may need to offer such protection to their parents. I have mentioned in the introduction that the parents themselves usually bear a certain amount of responsibility for ensuring that they do not become or remain lonely even if the more indigent they are, the smaller this amount will be. Other agents with duties to protect them from loneliness might include possible romantic partners, siblings, friends, neighbors, and fellow care home residents. An investigation into what forms of loneliness protection these individuals ought to provide, or may need to provide, has to await another occasion.

## Footnotes

1 Which will depend upon e.g. the strength of their filial duties, the physical distance between them and their parent(s), and whether such visits render it difficult for them to discharge moral duties towards other individuals, such as ones towards any children of their own.

2 This is true at least within contemporary Western countries on which this article focuses. Within societies with harsher living conditions, the relevant cut-off will be lower as people will experience faster physical and cognitive decline.

3 Who might include, *inter alia,* any children, friends, romantic partners, siblings, and neighbors that they might have.
